# Changes in chronotype and social jetlag during adolescence and their association with concurrent changes in BMI-SDS and body composition, in the DONALD Study

**DOI:** 10.1038/s41430-021-01024-y

**Published:** 2021-10-26

**Authors:** Nicole Jankovic, Sarah Schmitting, Bettina Krüger, Ute Nöthlings, Anette Buyken, Ute Alexy

**Affiliations:** 1 Nutritional Epidemiology, DONALD Study, Department of Nutrition and Food Sciences, Rheinische Friedrich-Wilhelms-University Bonn, Bonn, Germany; 2 Institute of General Practice and Family Medicine, Faculty of Medicine, Ruhr-University Bochum, Bochum, Germany; 3 Faculty of Natural Sciences, Institute of Nutrition, Consumption and Health, Paderborn University, Paderborn, Germany

**Keywords:** Risk factors, Epidemiology

## Abstract

**Background/objectives:**

Adolescence is a critical period for both the development of overweight and the transition toward a later chronotype, often accompanied by an increase in social jetlag. This study assessed whether changes in chronotype and social jetlag, are linked to changes in body composition during adolescence.

**Subjects/methods:**

We used data from the DONALD open cohort study, collected between 2014 and 2019, from 213 adolescents (9–17 years at baseline, 45% females) having at least two measures of chronotype and anthropometry (*N* = 572). Chronotype was assessed with the Munich Chronotype Questionnaire and defined as: midpoint of sleep corrected for sleep-debt (MSFsc) accumulated over the week (later MSFsc represents later chronotype). Social jetlag (SJL) defines the difference between midpoint of sleep during week and weekend. Calculations for Fat Free Mass Index (FFMI [kg/m^2^)]) and Fat Mass Index (FMI) [kg/m^2^)]) were based on body fat percentage, weight, and height. To analyze the associations, we used linear mixed-effect regression models. Finally, the total cohort was split into three biologically relevant age groups (cut-off set at <12 years, ≥12 to ≤15 years and >15 years).

**Results:**

Median follow-up was 2.1 years. Overall, change toward a later chronotype was significantly related with an increase in FMI (ß: 0.05, 95% CI: 0.01–0.08). A 1 h increase in social jetlag predicted an increase in BMI-SDS of 0.08 SDS units (95% CI: 0.01–0.14) and in FMI of 0.04 kg/m2 (95% CI: 0.003–0.08). Associations were stronger for the age group ≥12 to ≤15 years (*p* for interaction: <0.001). No relationship was found with FFMI.

**Conclusions:**

Changes in MSFsc and SJL during adolescence were associated with concurrent changes in BMI-SDS and FMI. The age ≥12 to ≤15 years appears to be a sensitive period in which chronobiological changes were clearly associated with increasing body fatness.

## Introduction

Chronotype describes the individual preference in sleep and wake timing [1]. Misalignment between the individual chronotype and socially determined sleep-wake-schedules result in “social jet lag”, meaning sleep deficits accumulate during the week and result in oversleep during the weekend [2]. Age and sex are important determinants regarding the individual chronotype [3]. During adolescence (age 9–18 years) a shift from an earlier to a later chronotype occurs [1]. More specifically the age of 12 years has been identified previously in a German population [4] to mark the start in eveningness, whereas the age of about 16 years represented the peak in lateness [5]. These cut-offs may slightly differ between populations [6]. Having a late chronotype and/or being socially jetlagged during adolescence are two important chronobiological factors, associated with a higher BMI [7–9]. In addition, adolescence is considered a critical period for the development of obesity and unfavorable changes of other body composition measures e.g., Fat Mass Index (FMI), likely tracking into adulthood [10, 11].

Hence, our hypothesis was that changes toward later chronotype or an increased social jetlag (SJL) during adolescence are associated with concurrent detrimental changes in body composition that might differ across age groups. We used repeated measurements of the DONALD Study over a median time span of 2.1 years (Q1–Q3:1.9–3.3) to define change in chronotype or SJL and body compositional changes of BMI-SDS, FMI and FFMI.

## Materials and methods

### Study design

The DONALD study is an ongoing, prospectively designed open cohort study conducted in Dortmund, Germany. Since 1985, data on diet, growth, developmental and metabolic factors are continuously collected from infancy (age 3 months) to adulthood. Approximately 30–35 healthy infants from Dortmund and surrounding communities, whose mothers and/or fathers have a sufficient level of the German language, are recruited every year either via personal contacts, maternity wards, or pediatric practices. The examination schedule includes quarterly examinations in infancy, half-yearly examinations in the 2nd year of life and annual examinations thereafter until young adulthood. Examinations include anthropometric measurements, lifestyle questionnaires and a 3-day food record. Chronotype and SJL assessment in the DONALD study started in 2014 for participants from 9 years onwards by use of the Munich Chronotype Questionnaire (MCTQ) [1, 12]. Detailed information regarding the study design can be found elsewhere [13]. Participants themselves or their parents gave written informed consent. The Ethics Committee of the University Bonn, Germany approved the study.

### Study sample

Among the participants of the DONALD study, those adolescents (aged 9–18 years) with at least two completed MCTQs and parallel collected anthropometric measurements were selected. From 2014 until July 2019, hence considering 5 years of follow-up, *n* = 620 study participants completed the MCTQ several times during the study (*N* = 1163). Those questionnaires collected during the 2 weeks after the time change in Germany from standard to summer time or vice versa [14] were excluded (*N* = 89). 378 questionnaires were completed during adulthood (i.e., 18 years and above) leaving 696 (*n* = 311) completed questionnaires during adolescence. Two participants with incomplete questionnaires (*N* = 3) were also excluded from the dataset. Anthropometric measurements within 1 year before or after completing the MCTQ were missing for 35 questionnaires (*n* = 10 subjects).

**Fig. 1:**
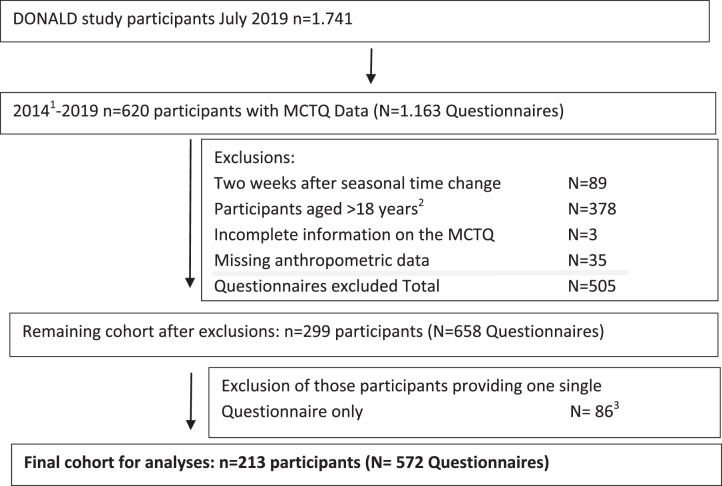
Study Flow Diagram for participant data from the DONALD Study. ^1^Munich Chronotype Questionnaire Assessments (MCTQ) started in the DONALD Study. ^2^Participants above the age of 18 years were considered as being adult. ^3^Necessary exclusion criteria for the overall analyses, evident after the assessment of model assumptions.

Hence, the final sample consisted of 213 adolescents (*n* = 95 females, 45%) out of 181 families providing 572 questionnaires (Fig. 1).

### Chronotype and social jetlag

The MCTQ includes questions regarding sleep and wake times during the week and weekend. The individual chronotype (continuously in h:min) was calculated as the Midpoint of Sleep (MSF), i.e., the half-way point between sleep-onset and sleep-end on free days [12] corrected for “oversleep” on free days (MSFsc) to account for sleep debt accumulated over the week [1]. The absolute value for social jetlag (SJL, continuously in h:min) was calculated as the difference between midpoint of sleep during free- and schooldays [7].

### Anthropometric measures

Adolescents were measured annually by trained nurses according to standard procedures, dressed in underwear and barefoot. Standing height is measured to the nearest 0.1 cm using a digital stadiometer. Weight was measured to the nearest 0.1 kg with an electronic scale (model 753 E; Seca, Hamburg, German). Skinfold thickness was measured on the right side of the body at the biceps, triceps, subscapular, and suprailiac sites to the nearest 0.1 mm with a Holtain caliper (Holtain Ltd., Crymych, UK) [13]. Sex- and age-independent body mass index (BMI kg/m²) standard deviation scores (SDS) were calculated using the German national reference data according to the LMS Method [15]. Percent body fat (%BF) was estimated from two skinfolds (triceps, subscapular) using age specific Slaughter equations [16], for the subsequent calculation of FMI (fat mass/m²; where fat mass = body weight * BF%/100) and FFMI (fat free mass/m²; where fat free mass = body weight − fat mass). Since the distribution of FMI was skewed, log10-transformed values were used in the analyses.

### Assessment of covariates

Parents are interviewed regarding family characteristics (i.e., parental education, persons per household). Every 4 years maternal body weight and height were measured with the same equipment as for the children on the child’s admission to the study center.

Adolescents’ pubertal status was assessed in different ways: Tanner stage (breast and penis development) [17] was visually assessed by the study physician at each visit after the age of 5 years. After Tanner stage 2, boys self-assed Tanner stage in case they considered the visual inspection as intrusive. In addition, age at take-off (ATO), Age at Peak Height Velocity (APHV), i.e., the age at minimal (ATO) and maximal (APHV) height velocity (zero acceleration) representing the onset and end of puberty growth spurt. ATO and APHV were derived from the parametric Preece and Baines Model 1 (Preece and Baines, 1978), details are explained elsewhere [18, 19].

Individual total energy intake was calculated from weighed dietary records as individual means of three record days [14], collected in the weeks after anthropometric measurement. Physical activity was assessed [13] by an interview based questionnaire at the study center asking about the participation in organized and non-organized sports. Energy expenditure was estimated by multiplying the mean duration of activity (hours/day) with the estimated basal metabolic rate (using age and sex depended algorithms according to Schofield [20], kcal/hour) and metabolic equivalents (MET, according to Ainsworth [21]. If information on covariates was not available for the same point in time at which MCTQ were administered and anthropometric measurements were taken, we considered covariate measurements taken within a 5-year time frame, while ensuring that these values were taken during adolescence (9–18 years).

### Statistical analyses

All statistical analyses of the present evaluation were performed using SAS^®^ procedures (version 9.4; Cary, NC, USA). The significance level was set at *p* < 0.05. The computer codes used for the analyses of the data during the current study are available from the corresponding author on reasonable request.

Linear mixed effects regression models (PROC MIXED in SAS), including both fixed and random effects, were used to analyze the association between change in MSFsc or SJL (Δ MSFsc, Δ SJL, both in h:min, subtracting baseline exposure from exposure at each year of assessment with 0 difference at first assessment) and concurrent change of Δ BMI-SDS, Δ FFMI or Δ log-FMI during adolescence (Change-on-Change-Models) [22, 23]. Mixed-effects regression models consider all available measurements per individual. Meaning the number of questionnaires per individual is allowed to differ [24].

Model building was driven by the log-likelihood criterion to define the final crude model. Covariates for model adjustment were selected according to known predictors of BMI, body composition, and chronotype or SJL [25, 26, 8]. From here, we drew a directed acyclic graph (DAG, Appendix Fig. 1) to identify minimally sufficient adjustment sets (msas) using a diagram representing the relationships among the identified variables [27], revealing age at baseline, time between first and subsequent measurements (basic model, details can be found elsewhere [23]), sex, ATO, number of persons in the household and maternal BMI (kg/m^2^) as important covariates. The joint inclusion of the msas represented the final model. To address potential biological differences by age arising from pubertal [27], chronobiological [3, 9] or behavioral [28] driven changes in the association between Δ MSFsc or Δ SJL and Δ body composition, we additionally stratified our data by age groups classified as: <12 years, ≥12 to ≤15 years and >15 years. The chosen cut-offs were identified in previous research [4, 5] as turning points in circadian changes. For the overall analyses we considered data from any single participant with at least two measurements to calculate the change from the first measurement taken per individual. This does not hold for the age group specific analyses. Age group <12 contains included 34 single measurements (18%) and age group ≥12 to ≤15 years included seven single measurements (10%). However, according to Jacobs et al. the change-on-change analyses is capable to capture this misbalance in the data [22]. Due to the open cohort design participants can occur in multiple age strata.

The Akaike Information Criterion and the Bayesian Information were used to select the correlation structure best describing the correlated nature of the data. Tests for effect modification were performed by the inclusion of interaction terms between chronotype or SJL, age (years) and sex (boys/girls).

To manage missing data we undertook multiple imputations, using the MI procedure in SAS and explored the pattern of missingness. A comparison of differences in baseline characteristics between those with complete and incomplete data has been performed (Appendix Table 1). We generated an imputed database containing five imputed versions to predict missing values for ATO (*n* = 58, 10%) and maternal BMI (*n* = 5, <1%). Regression results were combined using the MIANALYZE procedure in SAS. Final models were tested regarding multicollinearity, heteroscedasticity, and normal distribution of residuals.

We performed the following sensitivity analyses: inclusion of total energy intake and physical activity, education of the mother, season of chronotype assessment as well as early life factors e.g., mothers’ age at birth and weight gain during pregnancy. Furthermore, we excluded participants who used an alarm clock during the weekend (*n* = 59, 10%). Besides stratification according to chronological age, we applied stratification by puberty stages (Tanner <2 and Tanner ≥2, *p* for interaction <0.05) as suggested previously by Baird et al. [28]. Finally, we translated log FMI values in percent change for each one hour increase in the exposure variable ((exp(beta) – 1) * 100 [29]) and calculated the populations absolute change in FMI based on the population median FMI at baseline.

## Results

During a median follow-up of 2.1 years (Q1–Q3:1.9–3.3 years) adolescents aged 9–18 years completed 3 MCTQs (Q1–Q3: 2–3). Table 1 shows the sample characteristics at first and last assessment during 2014 and 2019. On the population level BMI-SDS increased by 600% (from a negative to a positive SDS) and FMI by 11% in comparison to the first measurement taken. Both the MSFsc and SJL increased over time by almost 39 min and 17 min, respectively, while sleep duration decreased by 45 min across the week. Family characteristics showed a high percentage of well-educated mothers with a normal body weight and a low percentage of smokers in the household.

**Table 1:** Characteristics^a^ of *n* = 213 adolescents (age 9–18 years, *N* = 572 questionnaires) at first and last measurement over a maximum of 5 years follow-up, DONALD Study, 2015–2019^b^.

Variable	First measurement	Last measurement
Female n (%)	95 (45)	
Follow-up (years)^c^	2.1 (1.9–3.3)	
Age (years)	12.9 (11.0, 15.0)	15.1 (14.6, 18.0)
Pubertal markers		
* Growth curves*		
Age at take-off (years)	9.7 (8.1, 10.6)	
♂	10.5 (9.8, 11.2)	
♀	8.0 (7.4, 8.7)	
Age at peak height velocity (years)	12.8 (11.3, 14.0)	
♂	13.6 (12.9, 14.6)	
♀	11.2 (10.5, 11.9)	
Anthropometric data		
BMI (kg/m^2^)	19 (17, 22)	20 (18, 23)
BMI-SDS (kg/m^2^)	−0.01 (−0.69, 0.76)	0.05 (−0.70, 0.80)
FMI (kg/m^2^)	4 (2, 5)	4 (3, 6)
FFMI (kg/m^2^)	15 (14, 16)	16 (15, 17)
Overweight (yes)^d^	33 (15)	28 (13)
Chronobiological variables (hh:mm)		
* MSFsc*	3:28 (3:02, 4:15)	4:07 (3:23, 5:00)
* Social Jetlag*	1:45 (1:15, 2:25)	2:02 (1:28, 2:45)
Sleep onset week	21:55 (21:12, 22:40)	22:28 (21:30, 23:10)
Sleep onset weekend	00:22 (0:01, 0:23)	00:04 (0:01, 00:23)
Total sleep duration week	08:25 (7:35, 9:10)	07:40 (7:00, 8:30)
Total sleep duration weekend	09:45 (8:50, 10:30)	09:00 (8:25, 09:55)
Wake up alone during weekend	182 (85)	186 (87)
Season of chronotype assessment		
Spring	46 (21)	44 (21)
Summer	61 (28)	69 (32)
Autumn	56 (25)	49 (23)
Winter	50 (25)	51 (24)
Other risk factors		
Total Energy Intake (kcal/d)	1829 (1558, 2130)	1831 (1558, 2210)
Physical activity (kcal/d)^e^	328 (198, 562)	361 (207, 543)
Family characteristics		
Persons per household	4 (3, 4)	
Overweight mothers^f^	66 (31)	
Highly educated mothers^g^	160 (77)	
Smoking in the household (yes)	28 (13)	

*BMI* Body mass index, *SDS* Standard Deviation Score, *FMI* Fat Mass Index, *FFMI* Fat Free Mass Index.

^a^Characteristics are presented prior imputation. The following variables had (*n*) missing values ATO (58), Tanner (127), Energy (72), physical activity (128), persons per household (121), maternal BMI (5), maternal education (10).

^b^Results are presented as number and (percentage), median (25th, 75 h percentile) or mean ± Standard Deviation.

^c^Follow-up was calculated as time between last and first measurement_._

^d^Overweight definitions according to the International Task Force BMI cut-offs for children corresponding to an adult BMI of 25 kg/m^2^.

^e^Expressed as energy expenditure in kcal based on the sum of organized and unorganized sport activities.

^f^≥ 25 kg/m^2^.

^g^≥ 12 years of schooling.

After adjustment, greater Δ MSFsc was associated with a 5% increase in FMI kg/m^2^ over time (*β* = 0.05 (0.01, 0.08), *p* = 0.01). Considering the median FMI at baseline as the reference, this estimate would translate into a 0.2 kg/m^2^ increase in FMI due to increasing lateness in chronotype during adolescence. Change in SJL was associated with BMI-SDS as well as log FMI. Associations for BMI-SDS and FMI were rather robust concerning confounder inclusion (Table 2) but not for FFMI. Stratified analysis by chronological age (*p* for interaction <0.001) revealed that the overall association reported for the complete sample including all adolescents aged 9–18 years is confined to the age group of 12–15-year-old adolescents (Table 3). No significant associations were seen in any of the other age groups. Of note, changes in MSFsc, SJL as well as changes in BMI-SDS, FFMI and FMI were greatest for the age group ≥12 to ≤15 years in comparison to the other age groups (Appendix Figs. 2 and 3). Stratified analyses according to Tanner stages (dichotomous with the cut-off at Tanner ≥2) showed an association during puberty only (data not shown). There was no indication for significant interaction of the examined associations with sex (Appendix Table 1).

**Table 2:** Regression coefficients of the linear mixed effects regression models for the association between change (Δ) in MSFsc or SJL and Δ BMI-SDS and Δ Body composition measures in *n* = 213 participants (*N* = 572 questionnaires) of, DONALD Study, 2015–2019.

	Δ MSFsc^a^			Δ SJL^b^		
Outcome of interest	beta	95% CI	*p* value	beta	95% CI	*p* value
Δ BMI-SDS						
Crude	0.06	(−0.01,0.13)	0.10	0.08	(0.02,0.15)	0.01
Model 1	0.06	(−0.01,0.14)	0,11	0.08	(0.01,0.14)	0.02
Δ FFMI						
Crude	0.18	(0.04,0.32)	0.02	0.29	(0.15,0.43)	<0.0001
Model 1	0.01	(−0.16,0.17)	0.92	0.06	(−0.10,0.19)	0.35
Δ log FMI						
Crude	0.05	(0.01,0.08)	0.01	0.06	(0.02,0.10)	0.002
Model 1	0.05	(0.01,0.08)	0.01	0.04	(0.003,0.08)	0.03

Crude: unadjusted model.

Model: 1 adjusted for age at baseline, sex, time between last and first measurement, age at take-off, persons in the household, maternal BMI (kg/m2).

*MSFsc* Midpoint of sleep, *SJL* Social Jetlag, *CI* Confidence Interval, *BMI-SDS* Body Mass Index-Standard Deviation Score (kg/m^2^), *FFMI* Fat Free Mass Index (kg/m^2^), *FMI* Fat Mass Index (kg/m^2^).

^a^BMI-SDS models contain a random intercept and slope for chronotype change with a variance components structure (VC). FFMI models contain a random intercept and slope for chronotype change and time with a VC structure. Log FMI models contain a random intercept and slope for time with an unstructured covariance structure (UN).

^b^BMI-SDS models contain a random intercept and slope for time with an un structure. FFMI models contain a random intercept and slope for time with a VC structure. Log FMI models contain a random intercept and slope for time with a VC structure.

**Table 3:** Results of the linear mixed effects regression models of the association between change (Δ) in MSFsc or SJL and Δ in BMI-SDS and Δ Body composition measures in *N* = 572 (*n* = 213) participants, stratified by age groups (years), DONALD Study, 2015–2019.

			Δ MSFsc^a^			Δ SJL^b^		
Outcome of interest^c^	*N*	*n*^d^	beta	95% CI	*p* value	beta	95% CI	*p* value
Δ BMI-SDS change								
Age <12	145	88	0.08	(−0.05,0.21)	0.24	0.03	(−0.10,0.17)	0.64
Age ≥12 ≤ 15	196	67	0.14	(0.02,0.26)	0.03	0.17	(0.07,0.30)	0.003
Age >15	231	58	0.06	(−0.06,0.17)	0.32	0.05	(−0.04,0.15)	0.27
Δ FFMI change								
Age <12	145	88	−0.02	(−0.29,0.24)	0.82	0.14	(−0.17,0.45)	0.31
Age ≥12 ≤ 15	196	67	0.08	(−0.08,0.25)	0.31	0.16	(−0.01,0.34)	0.06
Age >15	231	58	0.04	(−0.16,0.24)	0.70	0.02	(−0.19,0.24)	0.84
Δ Log transformed FMI change								
Age <12	145	88	0.02	(−0.05,0.10)	0.46	−0.02	(−0.11,0.08)	0.67
Age ≥12 ≤ 15	196	67	0.11	(0.05,0.16)	<0.001	0.12	(0.57,0.19)	0.001
Age > 15	231	58	0.02	(−0.03,0.08)	0.39	0.02	(−0.04,0.08)	0.42

*MSFsc* Midpoint of sleep, *SJL* Social Jetlag, *CI* Confidence Interval, *BMI-SDS* Body Mass Index-Standard Deviation Score (kg/m^2^), *FFMI* Fat Free Mass Index (kg/m^2^), *FMI* Fat Mass Index (kg/m^2^).

^a^BMI-SDS models contain a random intercept and slope for chronotype change with a variance components structure (VC). FFMI models contain a random intercept and slope for chronotype change and time with a VC structure. Log FMI models contain a random intercept and slope for time with an unstructured covariance structure (UN).

^b^BMI-SDS models contain a random intercept and slope for time with an un structure. FFMI models contain a random intercept and slope for time with a VC structure. Log FMI models contain a random intercept and slope for time with a VC structure.

^c^All models were adjusted for age at baseline, sex, time between last and first measurement, age at take-off, persons in the household, maternal BMI (kg/m^2^).

^d^N: total number of questionnaires, n: number of participants.

## Discussion

Our analyses confirm the association between MSFsc or SJL and body weight status and fat mass as observed in other studies. In addition, we could show the strongest associations between Δ MSFsc or Δ SJL and changes in overweight measures like Δ BMI-SDS and Δ FMI in the subgroup of adolescents aged 12–15 years. This specific age group appears to be a vulnerable life stage for body composition changes due to a shift toward a later chronotype or greater SJL, during adolescence.

Despite different study designs and applied methodologies, most previous studies reported an association between chronotype or SJL and body composition [5, 7, 8, 29, 30] with only few exceptions [31–33]. The evidence on chronobiology in relation to body composition is largest in adolescents but is mainly based on cross-sectional data.

To the best of our knowledge there is currently no comparable study that assessed change-on-change associations. However, among the longitudinal studies, Culnan et al. [34], investigated chronotype as a predictor of increased BMI in a group of 137 college freshman aged 18.3 ± 0.6 years over a period of 8 weeks. Participants having a late chronotype (based on the Morningness-Eveningness Questionnaire) had a significantly greater BMI gain in comparison to participants with an early or medium chronotype (unstandardized beta = 0.50 BMI points, 95% confidence interval [CI]: [0.04, 0.95], *p* = 0,034). Another study analyzed the relevance of long term exposure to later bedtimes, which may reflect a later chronotype, from adolescence to adulthood in 3342 participants (age 12–32 years) for changes in BMI [35]. In that study, later average workday bedtime by 1 h from adolescence to adulthood, was associated with an increase in BMI of 2.1 kg/m^2^ per 6 years. Regarding SJL, Zwart et al. [33] found no association with BMI over 1 year in 83 adolescents aged 16 years and older. Still, their result could be interpreted as being in line with our analysis, as in their sample the critical time window for a meaningful exposure [5] may have passed and adolescents from age 16 years onwards may already have experienced transition toward an earlier chronotype again, resulting in lower SJL, which we did also highlight in our supplementary material (Appendix Fig. 2). Differences to our study were the use of BMI instead of age and sex adjusted BMI values (e.g., *z*-scores), the length in follow-up, study population, sample size and differences in confounder adjustments (i.e., Zwart et al. did not adjust for confounding variables) and the use of a single question regarding bedtime instead of a validated questionnaire assessing chronotype and SJL.

Based on Roenneberg et al. [1] we would consider a person with a MSFsc from 5:00 am onwards as being late. A large proportion (>75%) of our adolescents is far away from that MSFsc and below the level of lateness found in other studies [25, 34], which may result in weaker associations with BMI-SDS in comparison to the associations found for SJL and BMI-SDS. SJL was already rather common at baseline in our study i.e., from the start of adolescence which has also been reported in previous studies [29, 36].

Many mechanisms are potentially involved in the associations between chronotype and body composition change (e.g., variable instead of constant sleep behavior during school and free days [37], shorter sleep [29], misalignment between the metabolic pathways and hormones [38], in particular ghrelin [39] and leptin [40], metabolic changes [7, 41] pro inflammatory state and cortisol [42] in combination with stress [43]). Carskadon et al. [44] even suggested pubertal changes e.g., due to the sensitivity of circadian rhythms to reproductive hormones [30], being responsible for the change in chronotype during adolescence. Puberty, as a period of greatest risk for body compositional changes induced by changes in chronotype, appears reasonable as most of the underlying mechanisms mentioned above, occur during the transition from childhood to adulthood [10, 30]. Also, dietary changes during the transition toward lateness may be involved in the association with body composition changes, especially starting at age 12 years. As shown previously in the DONALD study, a greater eveningness in caloric intake during adolescence showed a higher overall caloric intake from age 11/12 years onwards but not earlier [45]. Changes in FFMI due to changes towards a later chronotype were no longer discernable after confounder adjustment, presumably because most importantly age and sex related changes in muscle mass explain the crude associations seen with chronotype. By contrast the association with BMI-SDS and FMI increases were not explained by covariates.

A major strength of this study is the prospective, longitudinal design of the DONALD study with repeated measurements collected on the same individual. This rich data allowed the determination of change over time and unmasking the dynamic nature of MSFsc, SJL and body composition during adolescence. Further strengths of this study relate to the use of the MCTQ as a validated method [1] applicable to adolescents [6] and the objective anthropometric measurements by trained staff at the DONALD study center, the assessment of many high qualitative covariates including Tanner staging [30], as well as other puberty measures (ATO, APHV) that allowed several sensitivity analyses.

FMI and FFMI calculations are based on measured body weight and estimated body fat percentage derived from skinfold thickness. Even though body fat estimates, based on the slaughter equation, are fairly valid in non-obese (non-overweight DONALD adolescents about 87%) children [46], measurement error may still be a concern in terms of underestimating the true underlying association. However, the personal involved in the present study is trained regularly and thus, intra-observer variability and inter-observer variability are notably reduced [13]. Despite the possible limitations for FMI and FFMI, the use of FMI next to BMI-SDS allows for a better interpretation [47] and validation [48] of our results, that showed a selective increase in fat mass. Also, confounding by unknown or unmeasured covariates cannot be excluded. However, associations were rather stable regarding adjustment despite age and gender, which we considered in all adjusted models. Generalizability of our results may be limited since the DONALD study is characterized by a homogeneous sample of German participants, characterized by a high socioeconomic status. Even though, the prevalence overweight (15, 5%) is comparable to the overall German population (15, 4%) [49].

Finally, the amount of missing covariables was low (*n* = 63, 11%). Full case analyses would have likely biased our results differentially, meaning bias toward or away from the null. Therefore, multiple imputations were the best option to take care of missing data for the current analyses.

Our results contribute to the formulation of public health strategies regarding the risk reduction of overweight development during adolescence. Medical doctors should monitor adolescents carefully, especially from approximately age 12 years onwards, regarding changes in chronotype, since this change may contribute to circadian disruptions [41] leading to adverse effects on body composition [1, 26]. Targeted advice regarding good sleep practices, for instance consistent sleep timing or reduction of blue light exposure e.g., screen time before bed, appears promising [50–52]. Monitoring of bedtimes by parents may influence the sleep timing and therefore decrease the level of SJL [53]. However, since chronotype preferences seem to be a naturally occurring phenomenon another strategy is to delay school starting times [54]. If school is not delayed it would be advisable to maintain early bedtime schedules across the week and during the weekend to reduce behavioral as well as metabolic changes [7]. Future studies should consider stratification by puberty status or at least age to unmask a potential dilution of the overall association due to the dynamic nature of this population in terms of biological and physiological changes. So far, the direction of the association between chronotype or SJL and fat mass remains to be studied [55]. Therefore, the next step requires the analyses of chronobiological changes during adolescence and the impact on adult body composition which would help to clarify on the temporal ambiguity.

To conclude, changes in chronotype and SJL were associated with changes in body composition particularly with body fat during adolescence and between 12 and 15 years of age. Improvements of the social environment to support adolescents in line with their late internal clock, would be helpful to reduce their risk for unfavorable changes in body composition in this critical life stage.

## Supplementary information

Appendix Figures and Tables
