# Supplementary material for: Changes in chronotype and social jetlag during adolescence and their association with concurrent changes in BMI-SDS and body composition, in the DONALD Study
Source: Eur J Clin Nutr. 2021 Oct 26;76(5):765–71. doi: 10.1038/s41430-021-01024-y (PMC9090626; doi:10.1038/s41430-021-01024-y)
Supplement: Supplementary file 1 — Appendix Figures and Tables [file 41430_2021_1024_MOESM1_ESM.docx]

**Appendix Figures and Tables**

# Supplementary information

Figure 1 shows a simplified Directed Acyclic Graph for estimating the total effect of ∆ MSFsc or ∆ SJL on ∆ Body composition

Figure 2 and 3 (EXCEL) visualize the change in chronotype, social jetlag and body composition measures by age groups.

Appendix Table 1 includes the comparison of baseline characteristics, anthropometric and chronobiological variables in DONALD participants 2014-2019 who had complete anthropometric, chronotype and covariate data vs. those who did not.

Appendix Table 2 represents sex stratified regression coefficients of the linear mixed effects regression models for the association between change (Δ) in MSFsc or SJL and Δ BMI-SDS and Δ Body composition measures in n=213 participants (N=572 questionnaires) of the DONALD Study, 2014-2019

The final file is submitted as PDF.


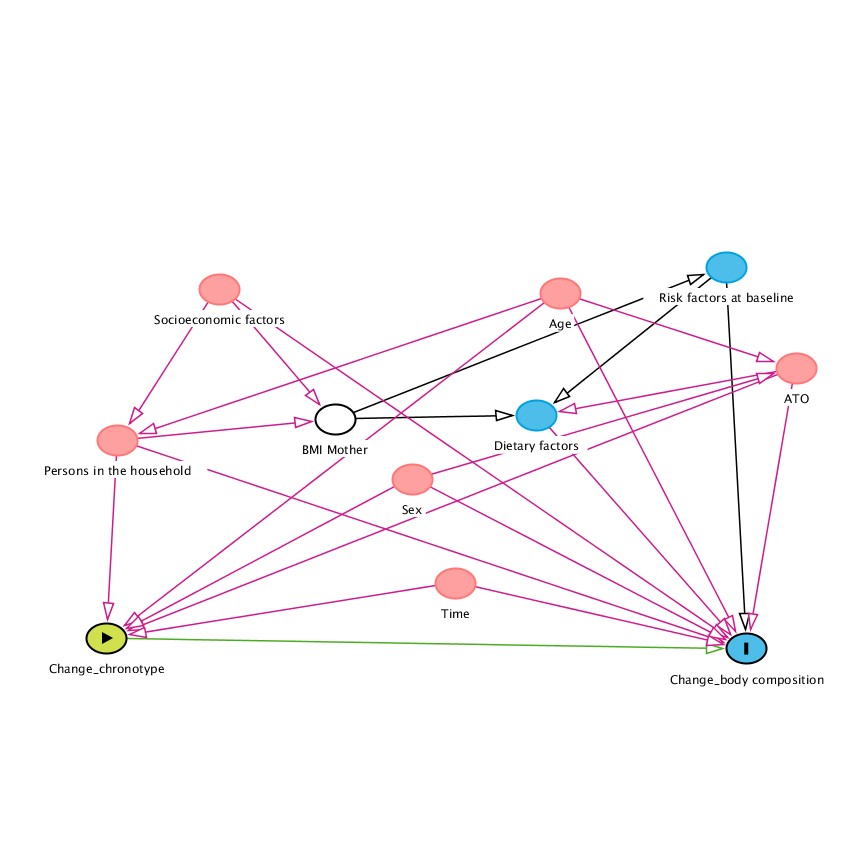


Appendix Figure 1. simplified Directed Acyclic Graph for estimating the total effect of ∆ MSFsc or ∆ SJL on ∆ Body composition.

Risk factors at baseline (physical activity, chronic diseases at baseline, sleep quality, birth markers), socioeconomic factors (maternal education and employment, smoking in the household), dietary factors (energy intake, alcohol intake, dietary pattern, season diet assessment, number of weekdays per diet assessment). The minimal sufficient adjustment set contained: ATO-Age at Take-off, Age, BMI Mother, Persons in the household, Sex, Time.

*p values were estimated with ANOVA

Appendix Figure 2. Median differences between the first and last measurement of chronotype assessed with the Munich ChronoType Questionnaire adjusted for oversleep during the weekend (MSFsc) and social jetlag in adolescents of the DONALD study 2014-2019, N 572 (213 adolescents).

*p values were estimated with Kruskal Wallice; ** p values estimated with ANOVA.

Appendix Figure 3. Median differences between the first and last measurement of body composition i.e. Body Mass Index standard deviation score (BMI-SDS), Fat Free Mass Index (FFMI) and Fat Mass Index (FMI) in adolescents of the DONALD study 2014-2019 N 572 (213 adolescents).

**Appendix Table 1** Comparison of baseline characteristics, anthropometric and chronobiological variables in DONALD participants 2014-2019 who had complete anthropometric, chronotype and covariate data vs. those who did not.

| **Characteristics of the DONALD Study participants** | **Eligible DONALD cohort** | | | | | |
| --- | --- | --- | --- | --- | --- | --- |
|  |  | **Complete data** for any of the covariates included in the model | | **Incomplete data** for any of the covariates included in the model | | P-value^1^ |
|  | N total | N | n (%) or median (p25, p75) | N | n (%) or median  (p25, p75) |  |
| **Baseline characteristics** | | | | | | |
| Sex | 213 | 153 |  | 60 |  |  |
| Male |  |  | 86 (56) |  | 32 (53) |  |
| Female |  |  | 67 (44) |  | 28 (47) | 0.70 |
| Age (years) | 213 | 153 | 12 (11, 15) | 60 | 14 (12, 16) | 0.03 |
| **Anthropometric data** | | | | | | |
| BMI-SDS (kg/m²) | 213 | 153 | -0.1 (-0.7, 0.5) | 60 | 0.3 (-0.5, 1.1) | 0.05 |
| FMI (kg/m²) | 213 | 153 | 3.3 (2.3, 5.0) | 60 | 4.2 (2.6, 5.7) | 0.02 |
| FFMI (kg/m²) | 213 | 153 | 14.8 (13.9, 16.0) | 60 | 15.5 (14.1, 16.8) | 0.06 |
| **Chronobiolological variables (h:mm)** | | | | | | |
| MSFsc | 213 | 153 | 3:10 (2:49, 3:55) | 60 | 3:32 (3:00, 4:16) | 0.03 |
| SJL | 213 | 153 | 1:45 (1:12, 2:17) | 60 | 1:57 (1:18, 2:33) | 0.15 |
| **Other variables** | | | | | | |
| Total energy intake (kcal/d) | 181 | 133 | 1790 (1557, 2080) | 48 | 1839 (1579, 2108) | 0.57 |
| Physical activity (kcal/d)^2^ | 210 | 152 | 321 (195, 515) | 58 | 361 (203, 605) | 0.39 |
| **Maternal factors** | | | | | | |
| Overweight^3^ | 211 | 153 | 52 (34) | 58 | 14 (24) | 0.17 |
| Education^4^ | 209 | 152 | 116 (76) | 57 | 44 (77) | 0.89 |

Abbreviations: BMI - Body mass index, SDS – Standard Deviation Score, FMI – Fat Mass Index, FFMI – Fat Free Mass Index

^1^Chi-squared test for categorical variables and Kruskal-Wallis test for continuous variables

^2^Expressed as energy expenditure in kcal based on the sum of organized and unorganized sport activities

^3^≥ 25 kg/m²

^4^≥12 years of schooling

**Appendix Table 2** sex stratified regression coefficients of the linear mixed effects regression models for the association between change (Δ) in MSFsc or SJL and Δ BMI-SDS and Δ Body composition measures in n=213 participants (N=572 questionnaires) of, DONALD Study, 2014-2019


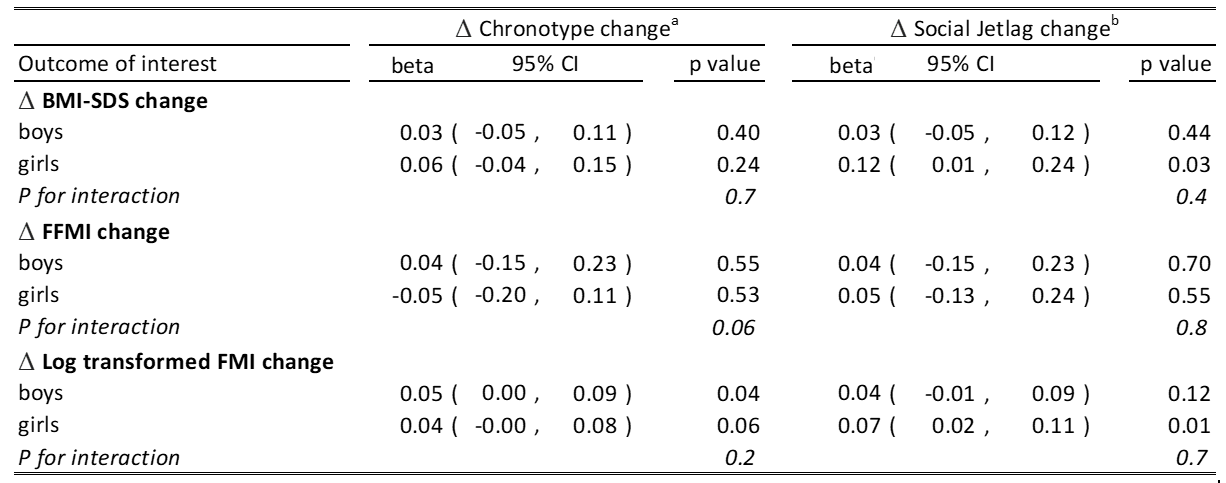


| Abbreviations: MSFsc – Midpoint of sleep, SJL – Social Jetlag, CI-Confidence Interval, BMI-SDS - Body Mass Index-Standard Deviation Score (kg/m^2^), FFMI - Fat Free Mass Index (kg/m^2^), FMI - Fat Mass Index (kg/m^2^). |
| --- |
| ^a^ BMI-SDS models contain a random intercept and slope for chronotype change with a variance components structure (VC). FFMI models contain a random intercept and slope for chronotype change and time with a VC structure. Log FMI models contain a random intercept and slope for time with an unstructured covariance structure (UN). |
| ^b^ BMI-SDS models contain a random intercept and slope for time with an un structure. FFMI models contain a random intercept and slope for time with a VC structure. Log FMI models contain a random intercept and slope for time with a VC structure. |
| Crude: unadjusted model. |
| Model: 1 adjusted for age at baseline, sex, time between last and first measurement,  age at take-off, persons in the household, maternal BMI (kg/m^2^). |
|  |
